# Microbiological diagnosis of the scalp and hair in 20–40 year olds – preliminary studies

**DOI:** 10.1515/biol-2025-1343

**Published:** 2026-07-14

**Authors:** Katarzyna Duda-Grychtoł, Karolina Oleś, Marta Palacz-Wróbel

**Affiliations:** Silesian College of Medicine in Katowice, Katowice, Poland; Pharmacology Department, University of Opole, Opole, Poland

**Keywords:** scalp microbiome, dysbiosis, trichological camera

## Abstract

Microbiome refers to the collection of microorganisms living in the human body. Skin, intestines and upper respiratory tract are particularly inhabited by microorganisms. The microbiota of the scalp and hair, though so far little understood, is particularly abundant in terms of microorganisms, which play an important role in maintaining the health of the scalp by inhibiting pathogens and promoting optimal skin condition. Dysbiosis of the scalp microbiota can be influenced by many factors, both exo and endogenous leading to the development of pathological conditions such as dandruff or seborrhoeic dermatitis. The aim of this preliminary study was the microbiological diagnosis of the scalp and hair in the group of individuals aged 20–40 years old. The samples for the research have been obtained from three different sites on the scalp of 6 individuals aged 20 to 40. The material for the study was collected using contact plates for total bacterial counts – Rodac Contact Test. The colonies grown on the microbiological media were characterised in terms of their size and shape. Subsequently, Gram staining was performed to assign the colonies of the bacteria to Gram-positive or Gram-negative bacteria, as well as fungi characterisation of the scalp and hair was conducted using a trichological camera. Finally, on the basis of the study following conclusions have been drawn. There were observed differences in the appearance of microbial colonies between younger and older people and in the group of 40-year-old, there was a significantly less colony diversity monitored. In terms of bacteria, Gram-positive cocci got isolated most frequently. In addition, yeast as well as filamentous fungi occurred abundant in the middle of the head of the research participants.

## 1 Introduction

Human skin is inhabited by commensal microbiota comprising bacteria, fungi, viruses, and demodex mites. The balance between all microorganisms inhabiting the skin is known as eubiosis. In contrast, dysbiosis refers to qualitative and quantitative disturbances in the skin microbiota, which can lead to numerous diseases. Studies on adults have demonstrated that the composition of the physiological skin microorganisms varies depending on the body site. The scalp is a favourable environment for microorganism development due to its numerous sebaceous glands. Although sebum possesses antimicrobial properties, certain microorganisms can survive in this environment [1]. *Cutibacterium acnes* as a one of the member of the skin microbiota is found predominantly in regions rich in sebaceous glands. *C. acnes* produces lipases metabolizing sebum and releasing free fatty acids in the pilosebaceous unit (PSU), in addition to several different triglyceride. Lipids have been shown to affect the ability of *C. acnes* bacteria to adhere to each other and to surfaces, and one of the functions of the *C. acnes* lipase appears to be enhancing colonization of the PSU by promoting the adhesion of cells to lipid-related components. The genome of *C. acnes* encodes at least 12 putative lipases. Lipases appear to play a determinant role in the growth of *C. acnes* in lipophilic environments. Their conformational structure may depend on the lipid level inside the PSU and could influence the pathogenicity of the strain. It is also worth emphasizing that *Cutibacterium* could suppress the overgrowth of *Staphylococcus* through the secretion of bacteriocin, while *Staphylococcus* owns an arsenal of different mechanisms to inhibit *C. acnes*, such as the fermentation of glycerol [2].

*Scalp is also heavily colonized by Malassezia. Malassezia* species are lipophilic and lipid-dependent yeasts. The interaction between *Malassezia* and the host is complex: it is usually symbiotic but can also be pathogenic. These basidiomycetous yeasts have been associated with dermatological diseases such as seborrheic dermatitis (SD), pityriasis versicolor (PV), atopic dermatitis (AD), and folliculitis decalvans (FD). This genus possesses the ability to encode multiple lipase genes, and the secretion of these lipases plays a significant role in the survival strategies of the host fungi and their pathogenic mechanisms. Lipases breaks down triglycerides and fatty acids in sebum, generating free fatty acids. These free fatty acids not only provide a carbon source for fungal growth but may also damage the host’s skin barrier [3], 4]. Data have shown that bacterial communities within hair follicles are dominated by genera such as *Propionibacterium*, *Staphylococcus*, and *Corynebacterium*. As it was shown before increased abundance of *Malassezia*, but also *Staphylococcus* and *Brevibacterium* are associated with SD. A higher proportion of *Actinobacteria* (includes *Corynebacterium*) and *Firmicutes* (includes *Staphylococcus, Streptococcus, Bacillus)* are present in alopecia areata (AA) patients. Decreased prevalence of *Actinobacteria* and increased *Staphylococcus* and *Streptococcus* are associated with scalp psoriasis [5], 6].

The scalp is constructed on the same principle as the skin of other parts of the body, but differs in a greater thickness of the dermis, hypodermis, the number of hairs and sebaceous and sweat glands. The influence of external factors on the scalp can change the microbiota, which will act as a catalyzing factor in the pathogenesis of skin diseases. Scalp health may be affected by physiological characteristics such as body mass index (BMI), sebum secretion, hair length, hormonal levels, scalp barrier function, and psychological and general health status, as well as daily hair care practices and lifestyle factors including shampoo functionality, washing frequency and the frequency of chemical treatments. Environmental exposures, including ultraviolet radiation and air pollution, living environment and climatic conditions also can influence scalp health [7].

Air pollution can promote oxidative stress and impair barrier function, and has been linked to reduced microbial diversity and a shift toward taxa associated with changes in TEWL (*Transepidermal Water Loss*) and skin hydration. In addition, UV radiation may reshape the microbial habitat by increasing oxidative stress, altering the local immune milieu, and stimulating cutaneous antimicrobial defenses, including the induction of antimicrobial peptide (AMP) expression in keratinocytes [7].

Psychological stress can increase the secretion of stress hormones, which impairs stratum corneum hydration, elevates TEWL, and alters the composition of epidermal lipids and structural proteins-ultimately leading to scalp barrier dysfunction as well as dysfunction of scalp microbiome. The use of cosmetic products is one of the major triggers of scalp dysbiosis. The use of hair dyes can induce scalp issues such as erythema, pruritus, desquamation, allergic reactions. In all these cases scalp dysbiosis may have existed. Further studies have shown that common ingredients in hair conditioners, such as preservatives (e.g., isothiazolinones), fragrances, and cationic polymers, may induce contact dermatitis, pruritus, and inflammatory responses by forming residues on the scalp and altering barrier function and the local microenvironment. Excessive cleansing can increase TEWL and impair barrier integrity, raising exposure to irritants and triggering scalp sensitivity [7]. Recent studies have suggested that incorporating probiotics or prebiotics into scalp care regimens may help restore balance and improve scalp health, indicating a promising avenue for future research and treatment [8]. Probiotics and its metabolites such as short-chain fatty acids (SCFAs), bacteriocins, exopolysaccharides (EPS), and exosomes have beneficial effects on skin and hair. SCFAs, such as acetic, propionic, and butyric acid, have immunomodulatory properties and can impact inflammatory responses. Bacteriocins produced by certain probiotic strains can inhibit the growth of harmful bacteria. EPS and exosomes enriched in microRNAs are involved in intercellular communication and may contribute to the modulation of immune and inflammatory processes. The most popular probiotics showed significant improvements for scalp and hair balance are *Lactobacillus paracasei* NCC2461, *Leuconostoc holzapfelii, L. mesenteroides, L. sakei, Rhynchosia volubilis Lour, Bifidobacterium lactis Bi-07 HN-019, L. acidophilus* NCFM, *L. rhamnosus* HN001, *L. paracasei* Lpc-37, *L. plantarum* TCI999, *B. lactis* CCT 7858 [9]. Postbiotics – non-living bacterial products or metabolites – are also emerging as therapeutic agents due to their anti-inflammatory, antioxidative, and microbiota-modulating effects. Kalibiome postbiotics from *L. paracasei* have demonstrated the ability to inhibit *Staphylococcus aureus* biofilms, reduce oxidative stress, and dampen inflammatory signaling, which may be relevant in scalp disorders and AA [10].

## 2 Materials and methods

### 2.1 Study participants

The material for the study has been collected from three different sites on the head of 6 individuals. All participants came the city of Tarnowskie Góry, in Upper Silesia, Poland. The characteristics of participants are presented in Table 1 (Table 1).

**Table 1:** The characteristics of study participants [own preparation].

Gender	Age	No. of samples	Place of samples taken	Characteristics of scalp and hairs
Female	20-year-old, student	No. 1 – front of the head No. 2 – middle of the head No. 3 – back of the head		Often dyes her hair red and experiences itching of the scalp
Female	25-year-old, student	No. 4 – front of the head No. 5 – middle of the head No. 6 – back of the head		Dyes hair blonde highlights, dry scalp
Female	30-year-old, an employee of an accounting office	No. 7 – front of the head No. 8 – middle of the head No. 9 – back of the head		Dyes her hair black, itching of the scalp, increased hair loss
Female	35-year-old, office worker	No. 10 – front of the head No. 11 – middle of the head No. 12 – back of the head		Natural curly, uncoloured hair
Male	35-year-old, office worker	No. 13 – front of the head No. 14 – middle of the head No. 15 – back of the head		Uncoloured hair, itching of the scalp, visible inflammation, flaky. In addition, itching of the chest, dry skin of the face and scalp
Female	40-year-old, office worker	No. 16 – front of the head No. 17 - middle of the head No. 18 – back of the head		Increased hair loss

**Informed consent:** Informed consent was obtained from all individuals included in this study, or their legal guardians or wards.

**Ethical approval:** The research related to human use has been complied with all the relevant national regulations, institutional policies and in accordance with the tenets of the Helsinki Declaration.

### 2.2 Microbiological solid media ready to use and culture method

Rodac Contact Test for total microbial count (no cat. PR-0090, Btl. Ltd, Poland) has been used with each plates pressed for 10 s on three selected parts of the head: the front of the head (above the forehead, one plate), the middle of the head (top of the head, one plate) and back of the head (the area behind the ear, one plate). The method was repeated in the same way for each participant. All plates were protected with parafilm, labelled and placed in a laboratory incubator CLW-15 (Pol-Eko, Poland) at 37 °C for 24–48 h.

### 2.3 Microscopic and macroscopic features of bacteria

#### 2.3.1 Colony morphology observations

After incubations there was a colony observed under a colony counter with a magnifying glass J-3 (Chemland, Poland). Subsequently, the following features of colonies: shape, margin, elevation, colour and texture were respectively analysed and the photos of colonies got scanned and stored.

#### 2.3.2 Gram staining

As described above, all the colonies have been cultivated on Contact Plates in order to check their cells morphology and the smears of bacterial suspensions in sterile 0.85 % NaCl got dried on objective slides and stained with classical Gram staining method [11]. Also, the cell morphology was monitored by a light microscope (OBS 6 firmy KAREN & SOHN GmbH, Berlin, Germany) using immersion oil.

### 2.4 Scalp videodermatoscopy

Scalp videodermotoscopy images have been acquired with the TrichoScope Polarizer HR Dino-Lite MEDL7HM (5 Megapixel resolution, up to 200× magnification). The built-in polarizer filter minimizes the glaring effect of the scalp/hair [12], 13].

## 3 Results

The preliminary investigations were carried out to characterize scalp and hair microbiome in the group of study participants aged 20–40.

### 3.1 Macroscopic and microscopic features of scalp microoganisms

The microorganisms were cultured on Rodac Contact Test for total microbial count at 37 °C for 24–48 h.

#### 3.1.1 Colony morphology

There have been four types of colonies monitored on Rodact Contact Test for total microbial count. They were yellow round colonies, with smooth or gently wavy edge whose texture was compact and superficial growth was observed. Some of the colonies had a mark of the center. The colour of the second type colonies was creamy, they were round, with superficial growth, their edge was smooth and whereas the texture was compact. Third in a row were small semi-transparent colonies with superficial growth, smooth edge and round shape. One additional type of colonies was observed for participant no 3 in the front and in the middle of the head. These colonies were big, orange with irregular edge and shape.

There were no differences between the types of colonies isolated from individuals no. 1, no. 2, no.3, and no. 4, all of which had a great number of semi-transparent colonies on the plate from back of the head. For individual no 5, 35-year-old man, numerous yellow colonies were observed in the front, middle and back of the head with only few semi-transparent colonies present on all plates. For individual no 6, 40-year-old woman only semi-transparent colonies and cream colonies were observed with only few yellow colonies present on the plate. All the results are listed in table 2 (Table 2).

**Table 2:** Colony morphology on Rodac Contact Test for total microbial count [own preparation].

Participant no 1, 20-year-old student, woman		
Front of the head	Middle of the head	Back of the head
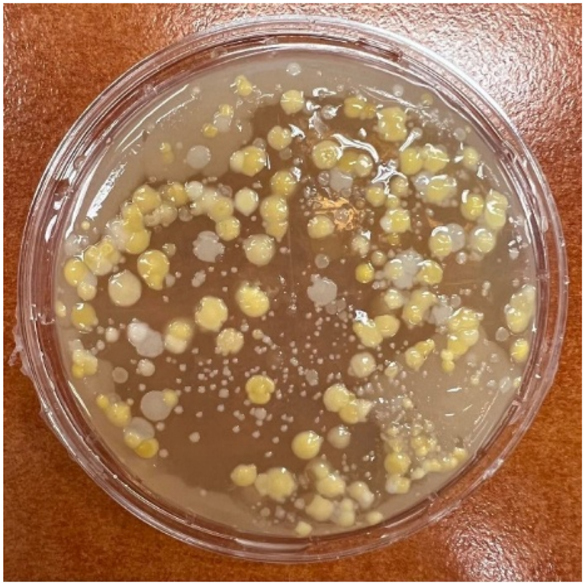 Total number of colonies: 656	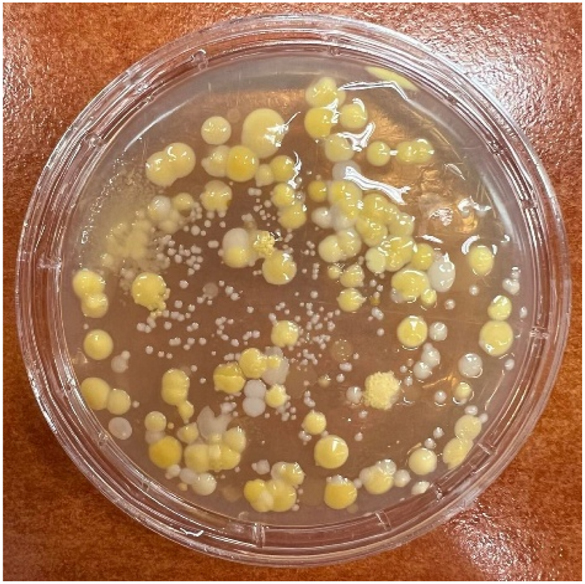 Total number of colonies: 436	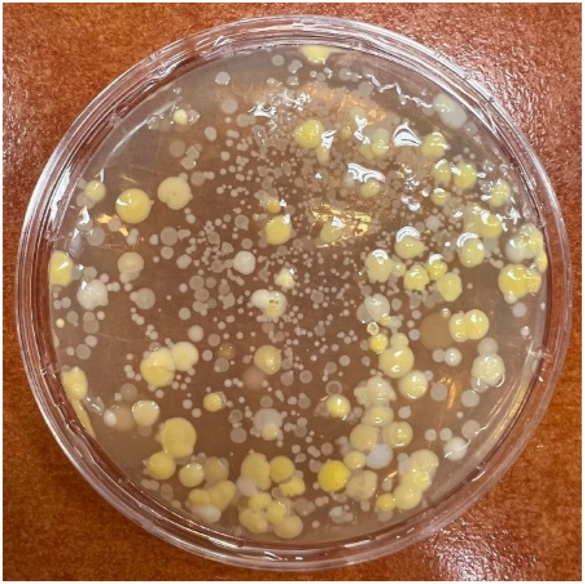 Total number of colonies: 809

Participant no 2, 25-year-old student, woman		
**Front of the head**	**Middle of the head**	**Back of the head**

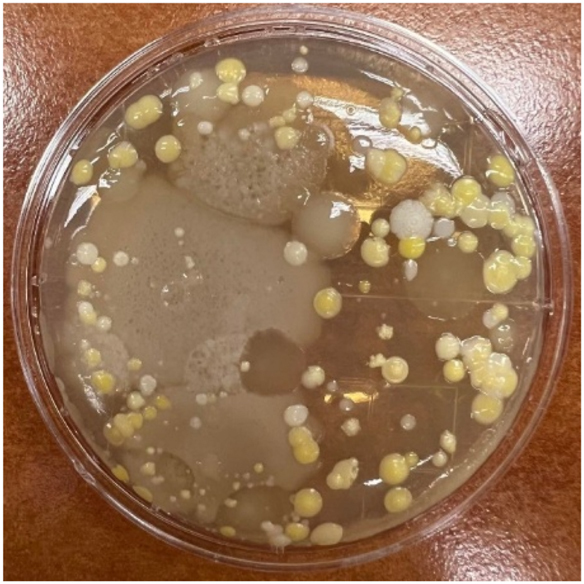 Total number of colonies: 194	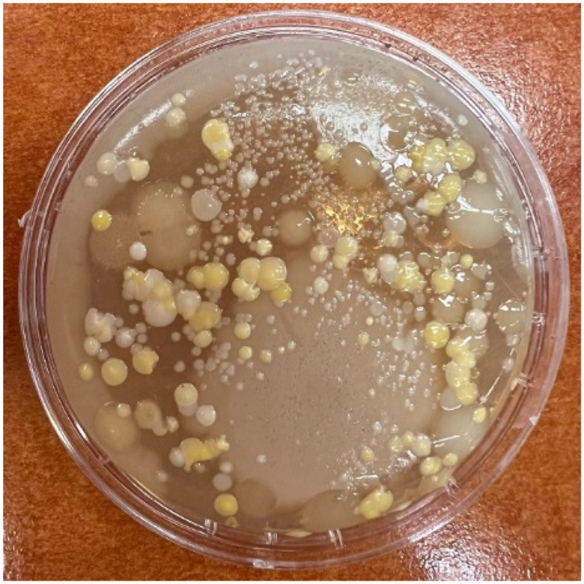 Total number of colonies: 684	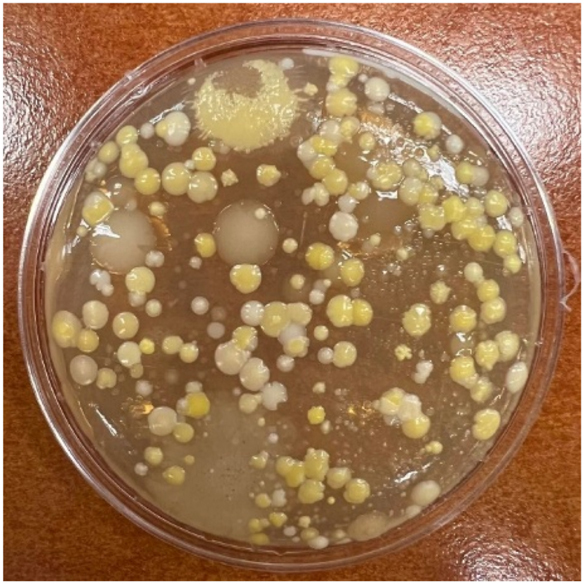 Total number of colonies: 220

Participant no 3, 30-year-old, an employee of an accounting office, woman		
**Front of the head**	**Middle of the head**	**Back of the head**

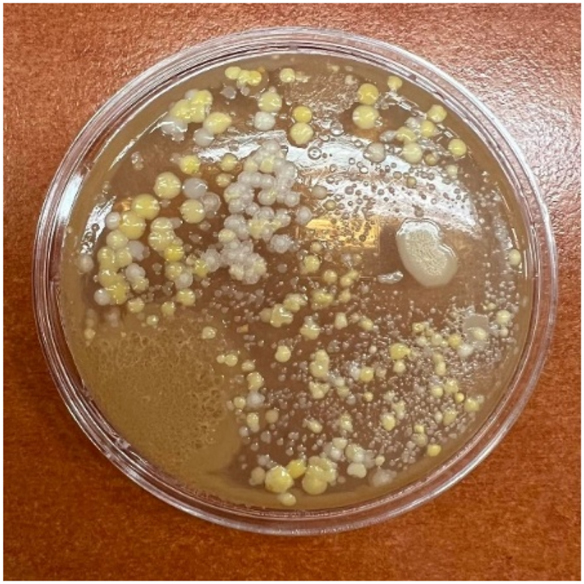 Total number of colonies: 505	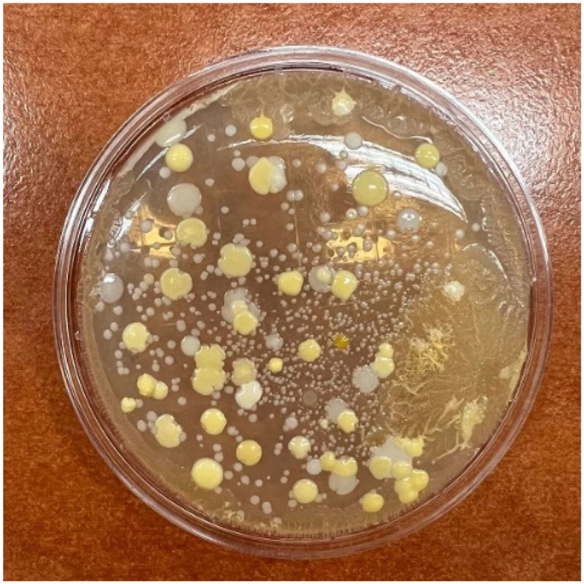 Total number of colonies: 458	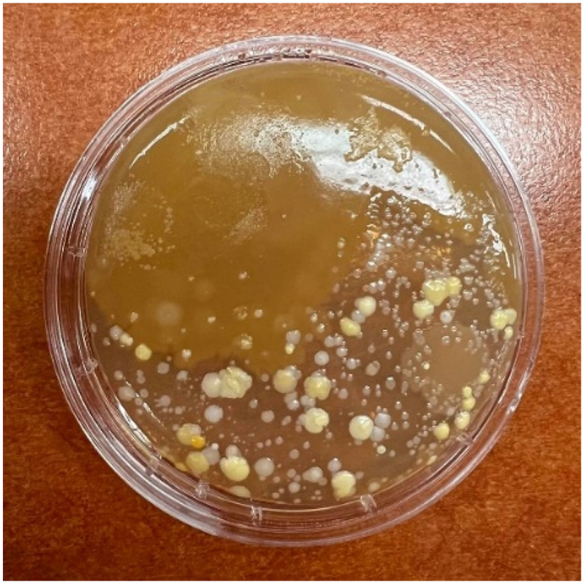 Total number of colonies: 281

Participant no 4, 35-year-old, office worker, woman		
**Front of the head**	**Middle of the head**	**Back of the head**

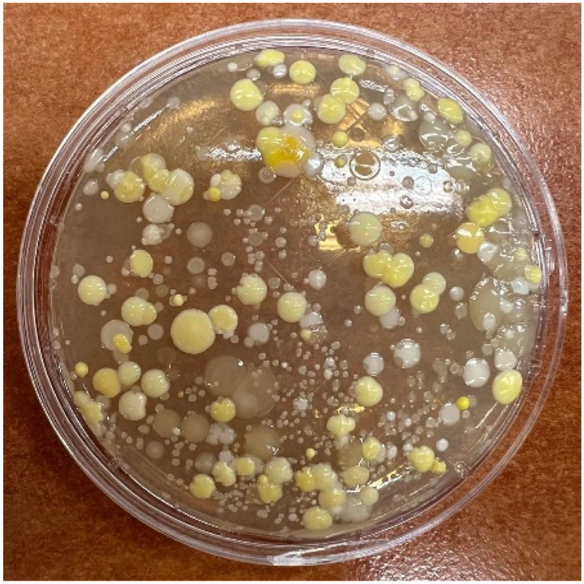 Total number of colonies: 454	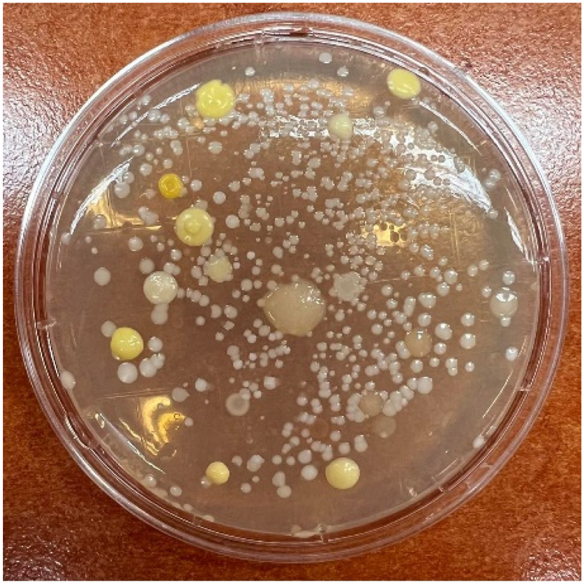 Total number of colonies: 404	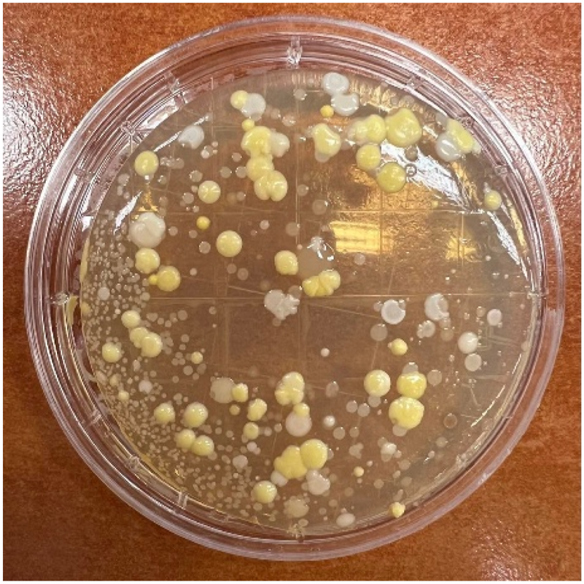 Total number of colonies: 394

Participant no 5, 35-year-old, office worker, man		
**Front of the head**	**Middle of the head**	**Back of the head**

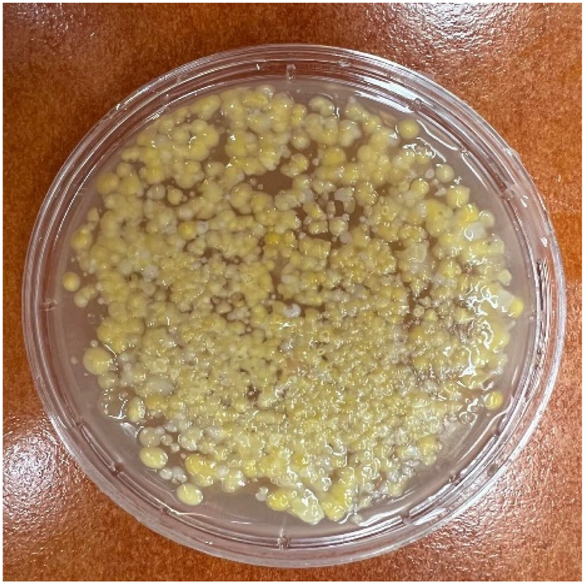 Total number of colonies: Uncountable	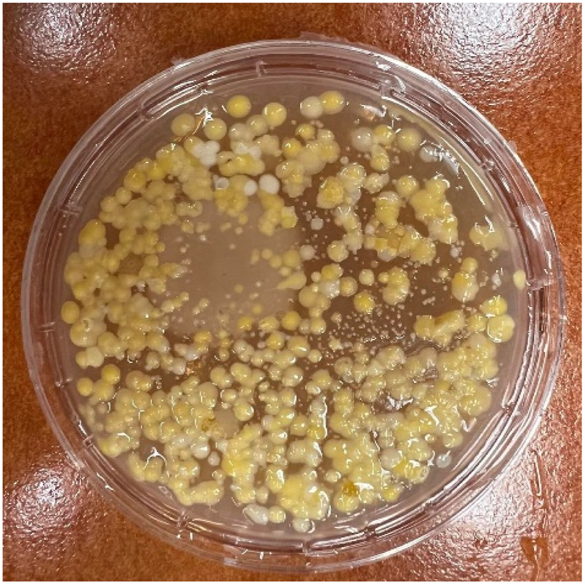 Total number of colonies: 675	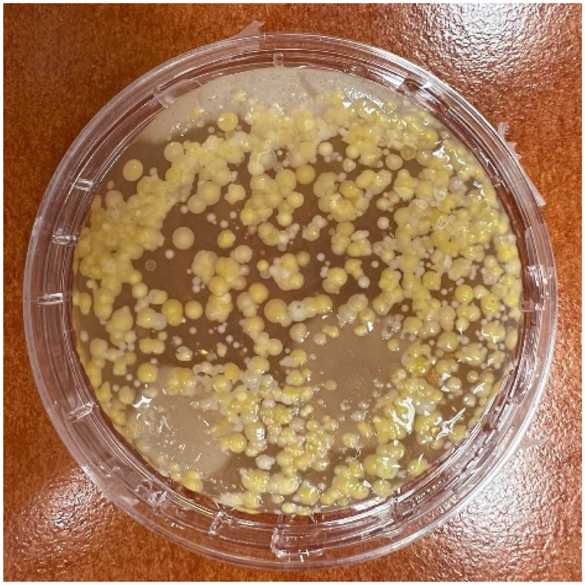 Total number of colonies: 625

Participant no 6, 40-year-old, office worker, woman		
**Front of the head**	**Middle of the head**	**Back of the head**

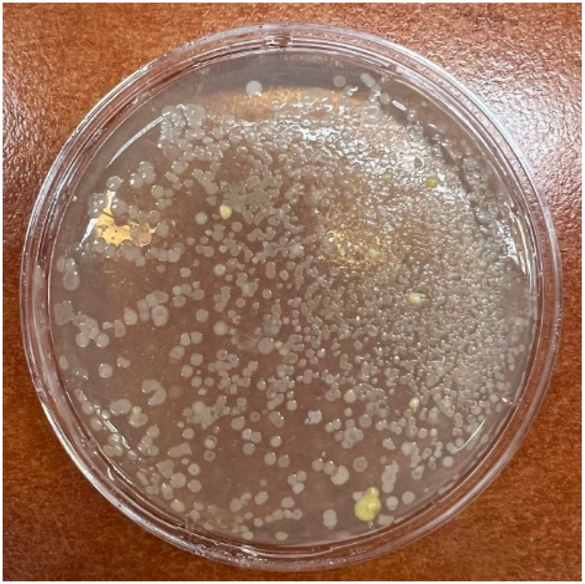 Total number of colonies: 683	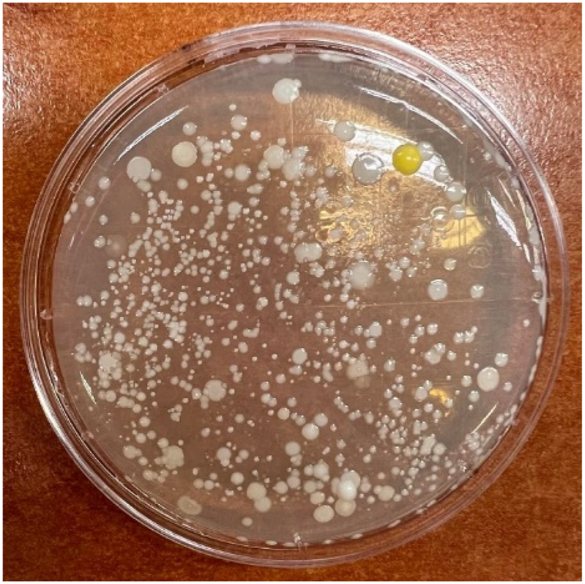 Total number of colonies: 402	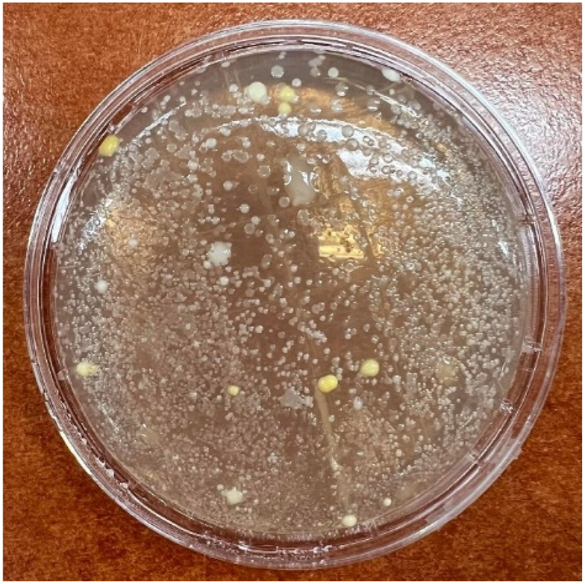 Total number of colonies: Uncountable

#### 3.1.2 Gram staining

Gram-staining of all colonies cultured on Rodact Contact Test medium at 37 °C for 24–48 h has shown that the obtained cultures contained Gram-positive, coccus-shaped and rod-shaped bacteria. For coccus – shaped bacteria micrococcus, diplococcic, and staphylococci were observed. For individual no. 4, there were yeasts observed in clusters and for individual no. 6 filamentous fungi with chlamydospores were observed. Selected examples of gram-staining photos are listed in Table 3 (Table 3).

**Table 3:** Selected examples of gram-staining colonies cultured on Rodact Contact Test media [own preparation].

Gram positive, cocci		
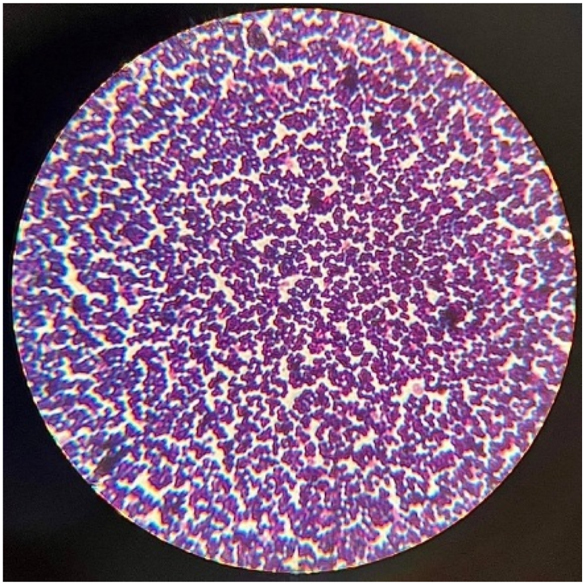 Arrangement: Staphylococci	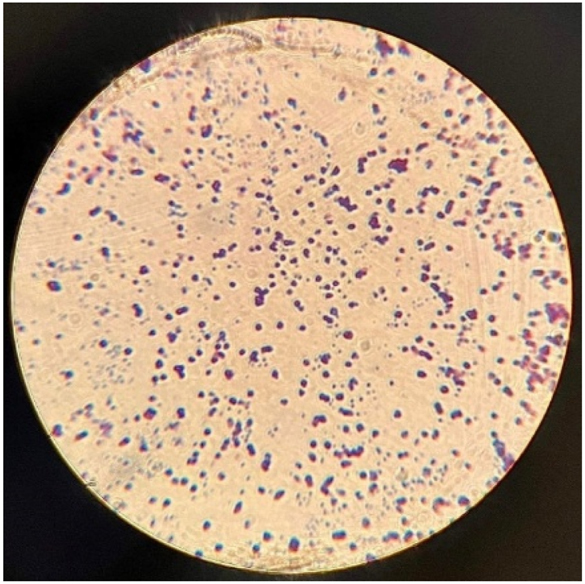 Arrangement: Micrococcus	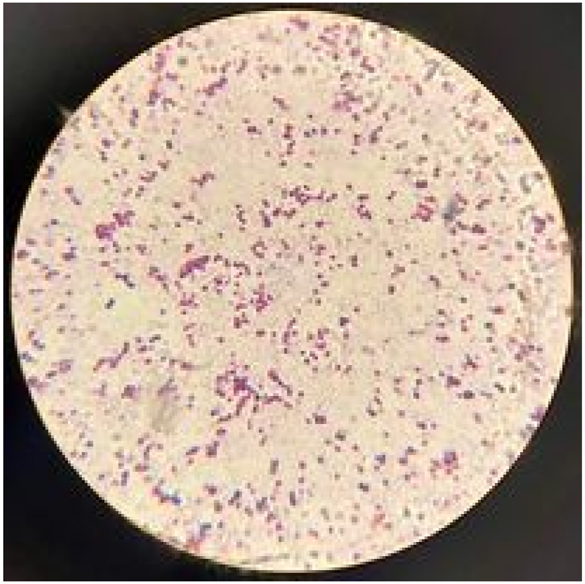 Arrangement: Diplococcus

**Gram positive, bacilli**		

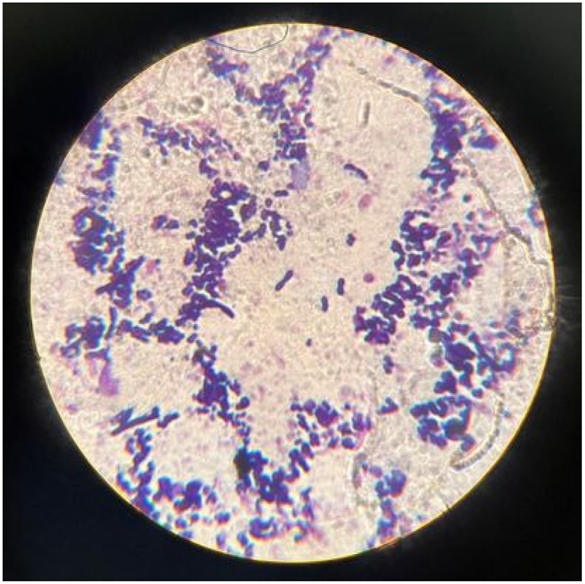 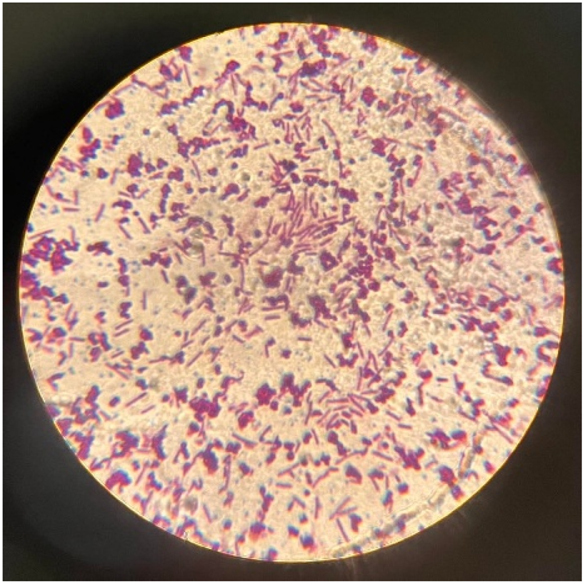 Arrangement: Rods **Filamentous fungi**		
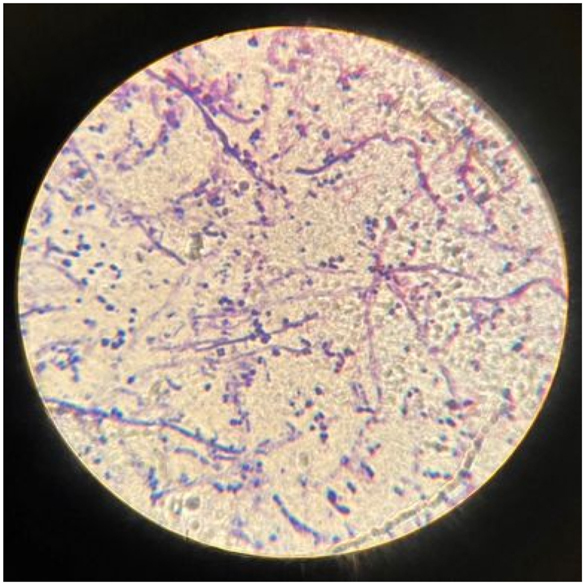 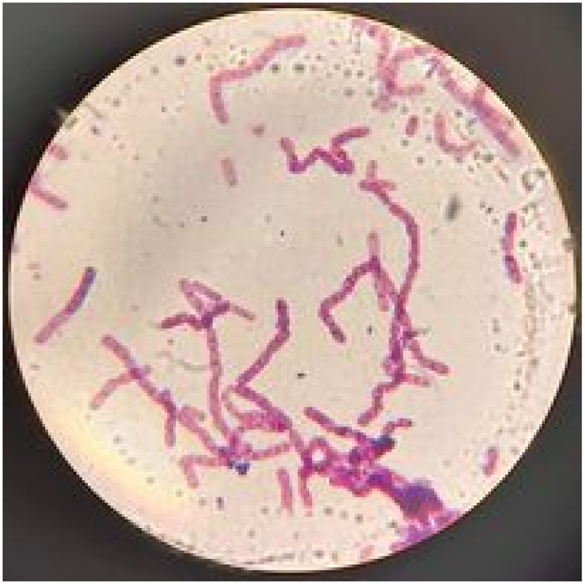 Arrangement: Hyphae, chlamydospores		

**Yeast**		

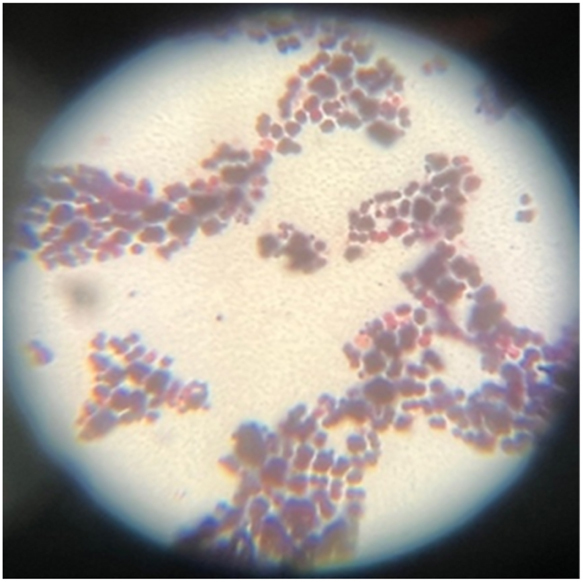 Arrangement: Cells of yeast		

### 3.2 Scalp and hairs videodermatoscopy

An analysis of trichoscopic images collected from study participants of scalp and hairs was conducted to develop scalp condition and hairs, and also some changes affected the skin balance microbiome. Results are listed in Table 4 (Table 4).

**Table 4:** Scalp and hair videodermatoskopy collected from study participants using TrichoScope Polarizer HR Dino-Lite MEDL7HM [own elaboration].

Trichoscopic images of scalp and hairs		
Participant no 1, 20-year-old student, woman	Participant no 2, 25-year- old student, woman	Participant no 3, 30-year- old, an employee of an accounting office, woman
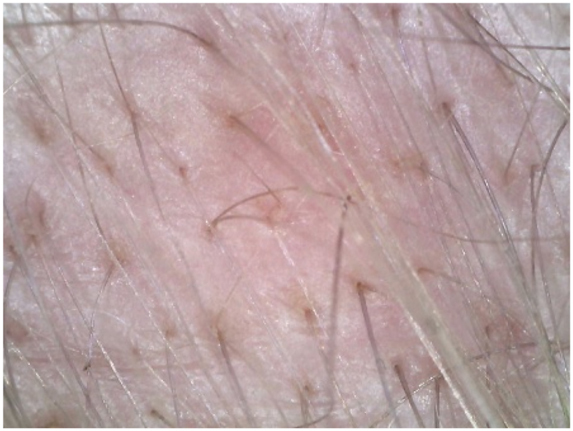 Detailed description: Spars, thin and light hair; skin pinkish	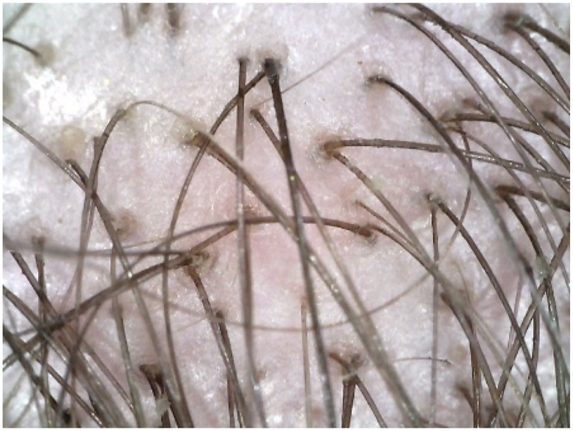 Detailed description: Plugged outlets of the pilosebaceous unit, visible layer of crystallization, greasy, dark hair	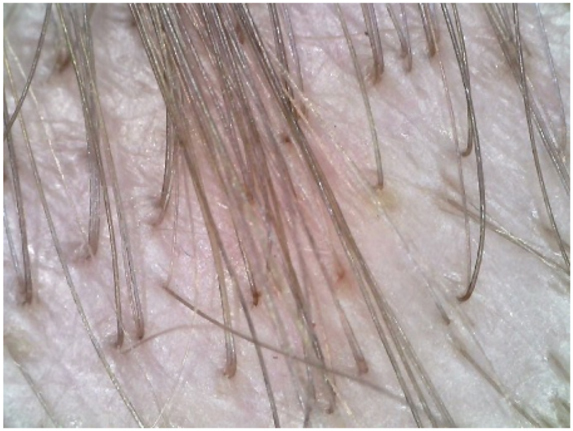 Detailed description: Light hair, pale skin with reddened spots, plugged outlets of the pilosebaceous unit

**Participant no 4, 35-year- old, office worker, woman**	**Participant no 5, 35-year- old, office worker, man**	**Participant no 6, 40-year- old, office worker, woman**

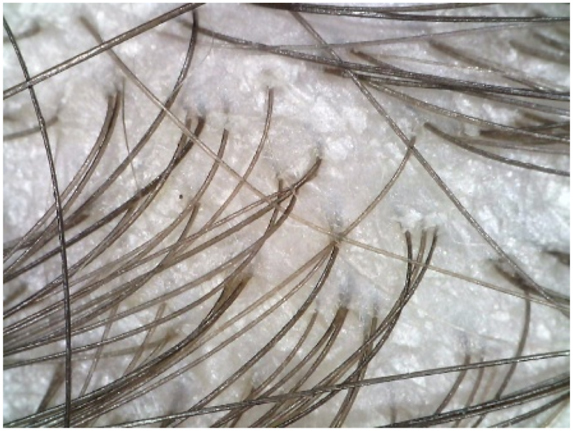 \ Detailed description: Plugged outlets of the pilosebaceous unit, scaly skin with flakes oily, dark hair	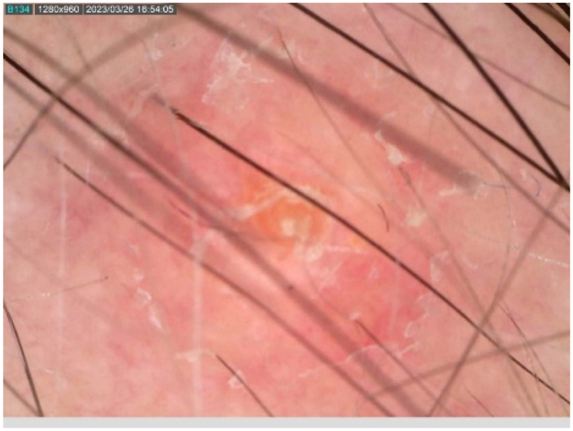 Detailed description: Hair miniaturisation, scaring areas, scaling, reddened skin, dark and pale hairs with different thickness, some hairs are broken	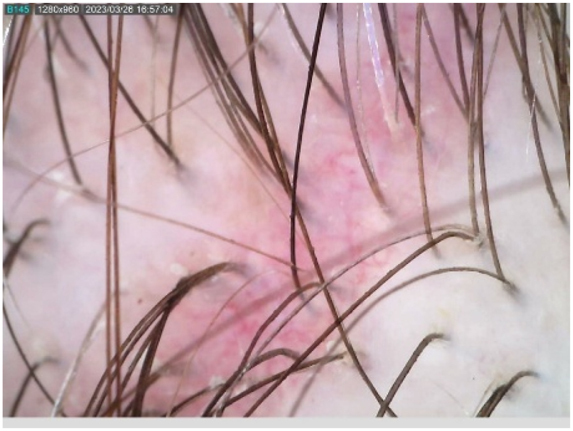 Detailed description: Reddened skin, visible serpentine vessels, dark and grey hairs, miniaturisation of individual hairs

## 4 Discussion

The scalp as well as other areas of the body is colonized by different types of microorganisms, which form the so-called physiological flora. The physiological flora is the natural microsystem responsible for maintaining the health of the body. The microorganisms populating the physiological flora of the human scalp include bacteria, viruses, fungi and archaea. The most commonly found bacteria in the physiological flora of this area include *Staphylococcus epidermidis* and *Cutibacterium spp*. Mycobiome of the scalp is mainly formed by *Malassezia spp* and *Ascomycota* filamentous fungi [14].

It is worth noting that the composition of the scalp flora can vary between individuals and its diversity is influenced by many factors such as age, diet, hygiene or environmental factors. Hence, the aim of the study submitted for evaluation was a brief microbiological diagnosis of the scalp and hair in individuals of different ages.

There were six individuals, aged between 20 and 40 years old selected for the research and the material for the study was gathered using Rodac Contact Test for total microbial count from three areas on the head. The colonies grown on solid media were then analysed, subjected to Gram staining and observed under a microscope. Also, the scalp of the participants was also examined with a trichoscopic camera.

Microbiological diagnosis of the individuals’ scalps has shown microbiological variation depending on the site from which the material was taken. Nevertheless, each participant revealed the presence of Gram-positive bacteria belonging to the cocci and cylindrical forms, which may indicate the presence of *S. epidermidis* and *C. acnes*. This result is corroborated by studies by Saxen et al. [15] as well as Clavaud et al. [16]. These studies showed that the scalp microbiome has a relatively low bacterial diversity compared to other sites on the body, and the most abundant bacterial groups found in scalp swabs from healthy individuals are *Cutibacterium spp.* with a predominance of *C. acnes* and *Staphylococcus spp*. with a predominance of *S. epidermidis*, accounting for about 90 % of the gene sequences. Other less abundant species include *Corynebacterium spp., Streptococcus spp., Acinetobacter spp.* and *Prevotella spp* [16]. A study by Matard on the deeper parts of the scalp follicle also showed the presence of Gram-positive bacteria. Images taken with field emission scanning electron microscopy and scanning confocal laser microscopy, showed structures consistent with bacterial biofilms beneath the hair follicles. These biofilms were composed of bacilli and morphologically corresponded to *C. acnes* [14].

The vast majority of studies on the fungal composition of the human scalp have shown the presence of *Malassezia sp* [17]. with a predominance of *M. globosa* and *M. restricta*. *Ascomycota* including *Acremonium spp.* and *Didymella bryoniae* and *Basidiomycota* including *Cryptococcus liquefaciens* and *C. diffluens* as well as *Coniochaeta spp., Rhodotorula spp*. have also been identified on the healthy scalp. A study by Jo et al. [18] showed that the mycobiom of children aged <14 years is more diverse, with a relatively lower proportion of *Malassezia* spp. compared to adults aged 20–30 years. This is most likely due to the result of lower sebaceous gland activity and differences in sebum composition before puberty [18]. In our study, in only two participants, who were the oldest, yeast and filamentous fungi have been identified. These microorganisms were isolated from the middle part of their head. This result is confirmed by the study by Shibagakil et al. [19], which was conducted on a group of women of different ages. The older group aged 60–76 had a greater diversity of species and a significant increase in minority species compared to the younger group (21–37 years). In our study, only in subjects aged 35 and 40 years was the presence of filamentous fungi observed, which were not isolated in the microbiome of younger individuals. These results may suggest that age may influence the composition and diversity of the scalp microbiome, although a much larger group of different ages would need to be studied to draw a definite conclusion [14].

One of the diagnostic methods used in the trichology practice is imaging diagnostics, and video dermatoscopes and trichology micro cameras. The analysis includes the entire scalp, both the healthy scalp and the affected areas, as well as the roots and shafts of the hair. Visual diagnosis can also be performed using a lamp – a magnifying glass – and the magnified image obtained allows the lesions on the scalp and hair to be observed. Another non-invasive analysis to assess the scalp is trichoscopy, which uses a digital video camera – a trichoscope. This method makes it possible to ascertain the condition of the scalp, the presence of telangiectasias and the condition of the hair follicles. Trichoscopy also makes it possible to assess the appearance and condition of the hair shaft, its thickness, pigmentation, shine or tendency to breakage [13], 20].

In our study, the TrichoScope Polarizer HR Dino-Lite MEDL7HM camera was used to analyse the scalp and hair. In two of six study participants (no 5 and no 6–35 and 40 years) we revealed scalp inflammation and hair changing. In these two cases yeast and filamentous fungi were observed. For a study participant no 5 there was much more bacteria growth on the plate in comparison to other participants as well as miniaturization of hair and scalp redness were noticed. For a participant no 6, the quantity of bacteria was smaller and bacteria colonies on the plates were less different compering to others and scalp redness, keratosis of hair follicles were present. In these two cases microbial dysbiosys as well as other changes may suggest presence one of the hair disorders such as seborrhea dermatitis or folliculitis. As mentioned before, bacterial communities within hair follicles are dominated by genera such as *Propionibacterium*, *Staphylococcus*, and *Corynebacterium.* These bacteria play pivotal roles in maintaining follicular health by modulating original vulnerable responses and precluding the colonization of pathogenic microbes. Fungal, particularly species of *Malassezia*, but also filamentous fungi are particularly abundant in sebaceous areas and are intertwined in conditions like dandruff and seborrhea dermatitis [21]. Filamentous fungi have not been diagnosed in 20-, 25- and 30-year-olds, nor has excessive keratinisation of the scalp. Dermatophytes, as they are known, are a group of filamentous fungi that have an affinity for keratin-rich tissues. Excessive keratin synthesis, therefore, will predispose to scalp colonisation by filamentous fungi. It is also worth mentioning that the effect on dandruff formation may largely be related to an insufficient amount of anaerobic *Cutibacterium* bacteria. Scaly skin with flakes was observed in study participants no 4, 35-year-old woman [22].

Furthermore, a trichoscopic examination in a 35-year-old individual (no. 5) showed a scaly scalp, which may suggest the presence of dandruff or other psoriatic conditions, and the significant keratinisation of the scalp, also present, may indicate excessive keratin production. Studies have shown that patients diagnosed with scalp dandruff have excessive colonisation of *S. epidermidis* and *Malassezia fungi – M. restricta* [22], 23].

Analysis conducted with a trichoscopic camera showed serpentine vessels in the study participant no 6 (40-year-old, woman), which may indicate a circulatory disturbance in the scalp, possibly related to the initial stage of alopecia [24].

In the case of 35-year-old office worker, man, lesions characteristic of seborrhoeic dermatitis were observed using a trichoscopic camera. The predisposition to seborrhoea is a genetic trait and dependent on the hormones androgens and progesterone, which stimulate sebum production. Seborrhoea is closely associated with hair loss, which is caused by blockage of the hair follicles by excess sebum, resulting in hypoxia and lack of blood supply to the hair follicle. Hair loss was one of the characteristics of the study participant numbered 5. The etiopathogenesis of SD is closely linked to the proliferation of lipophilic yeasts of the *Malassezia* species. Their metabolites such as fatty acids or indole-3-carbaldehyde can cause an inflammatory skin reaction. Despite the presence of *Malassazia fungi* in the natural physiological skin flora, their presence is particularly increased in cases of SD [3], 4], 22].

It is worth noting that advances in microbiome exploration methodologies, including metagenomics and high-throughput sequencing technologies play a crucial role in understanding a relation between hair follicle microbiota and its host. It has been shown that Gram-positive rods *Propionibacterium* modulate immune responses and prevent pathogen colonization. Gram-positive *Staphylococcus* (e.g. *S. epidermidis*) maintains skin barrier and modulates immune function. *Cutibacterium* (e.g. *C. acnes*) are involved in lipid metabolism and immune modulation. *Micrococcus* decomposes sweat, contributes to body odor. *Malassezia* e.g. *M. globosa* and/or *M. restricta* implicated in dandruff and seborrhea dermatitis. Various bacteriophages influence bacterial populations, potential roles in skin health. *Methanogens* e.g., *Methanobrevibacter smithii* involved in methane production and skin ecosystem balance.

Microbial alterations of scalp can lead to inflammation, immune responses, and structural damage to the hair follicles, thereby contributing to disease progression. In our research we have observed effects on the changes in microbion on scalp and hair condition depending on the people’s age. For sure using High-Performance Liquid Chromatography (HPLC) or metagenomics analysis performed using 16S ribosomal RNA gene sequencing are needed to complete results, as well as a larger study group [21].

## 5 Conclusions

The morphology of colonies grown on solid media was similar in all participants. Differences in the appearance of microbial colonies were observed between younger and older subjects. However, there was considerably less colony differentiation observed in 40-year-old individual. Microscopic analysis revealed the presence of predominantly Gram-positive cocci bacteria and Gram-positive rods. It can be suspected that these bacteria are mainly *Staphylococcus epidemids* and *C. acnes*. These results confirm the literature data. Yeasts and filamentous fungi were observed in two study participants (35-year-old man and 40- year-old woman). These microorganisms were isolated from the middle part of their head. The literature indicates that yeasts inhabiting the scalp are mainly of the genus *Malassezia*, while filamentous fungi include *Ascomycota*. Analysis of scalp using a trichoscopic camera revealed scalp lesions in participants no 5. These lesions may have suggested seborrhoeic dermatitis. Confirmation of this result, however, requires further careful analysis.
